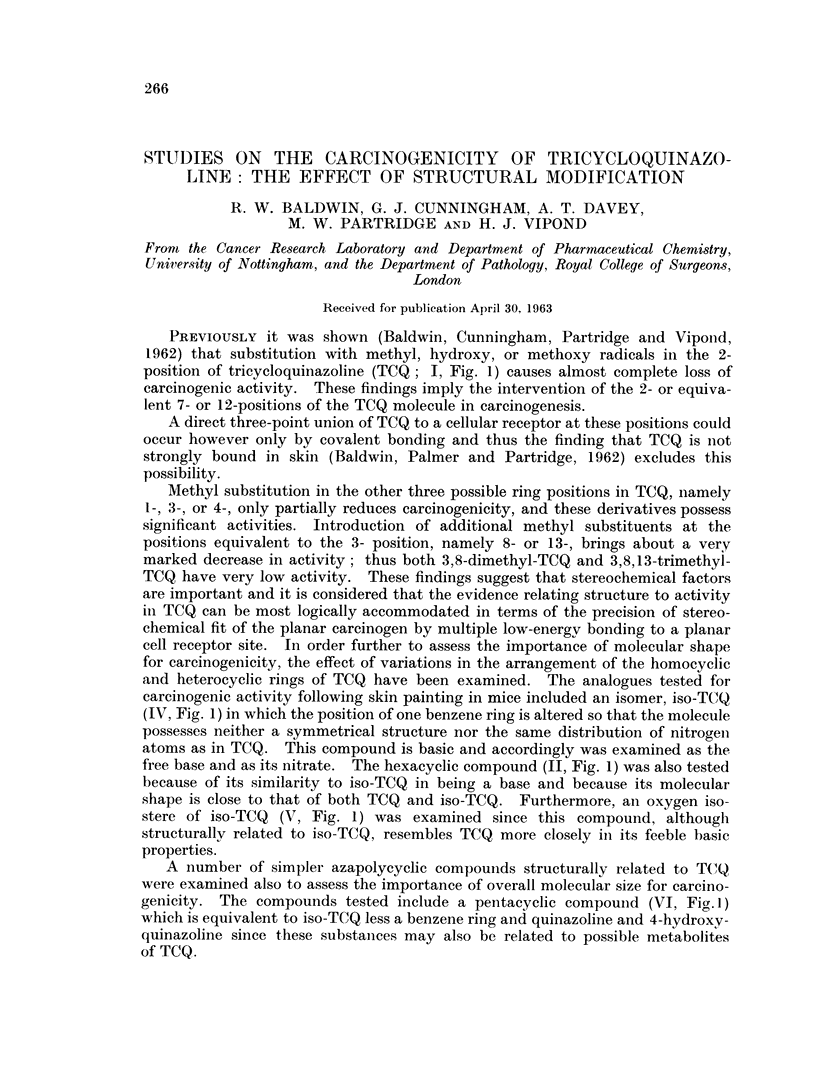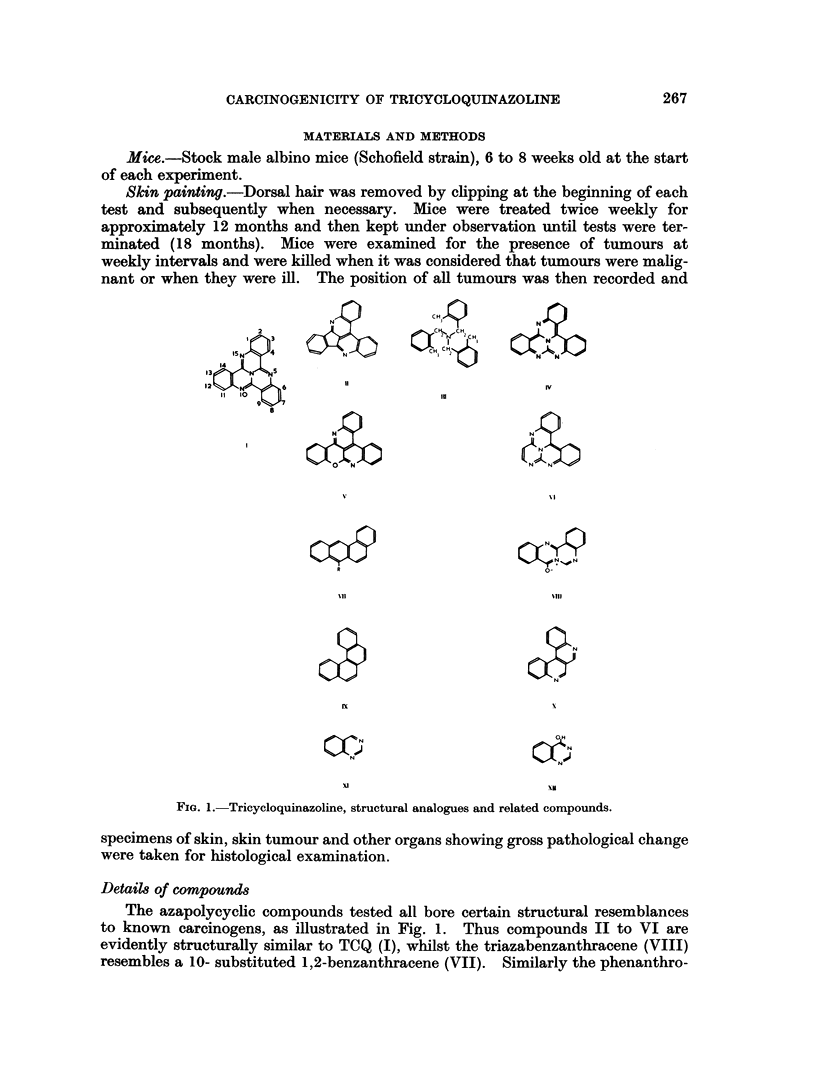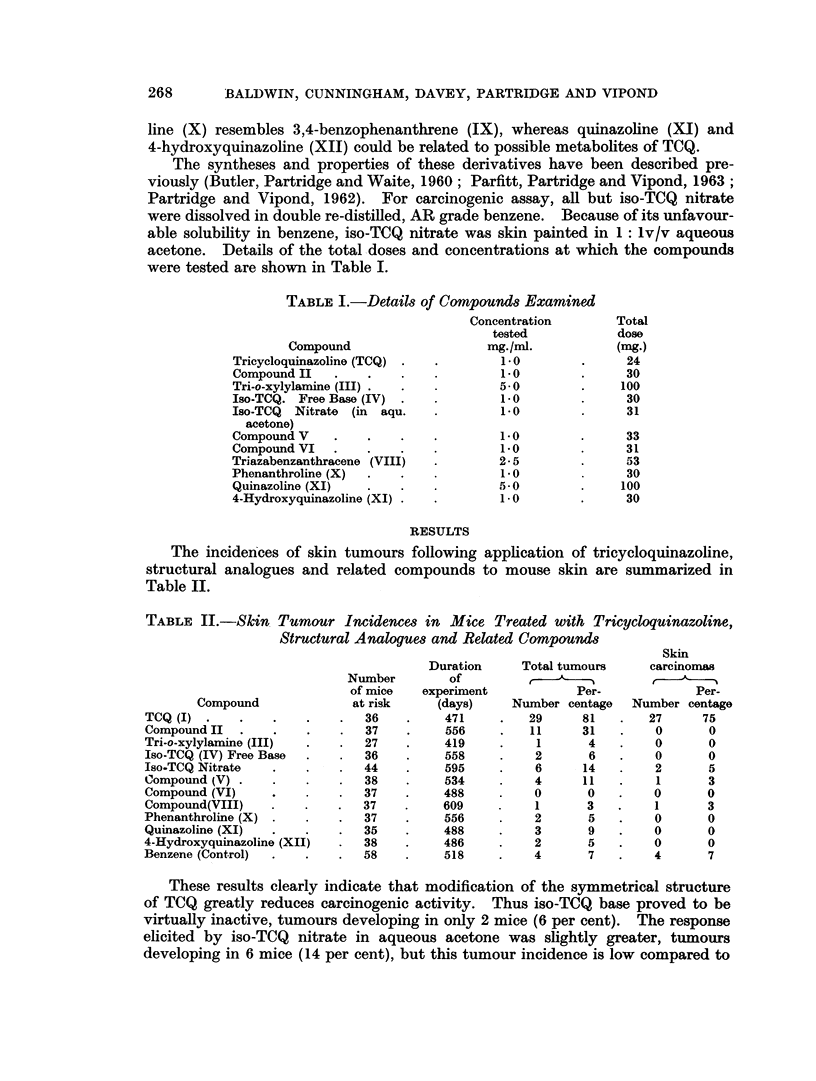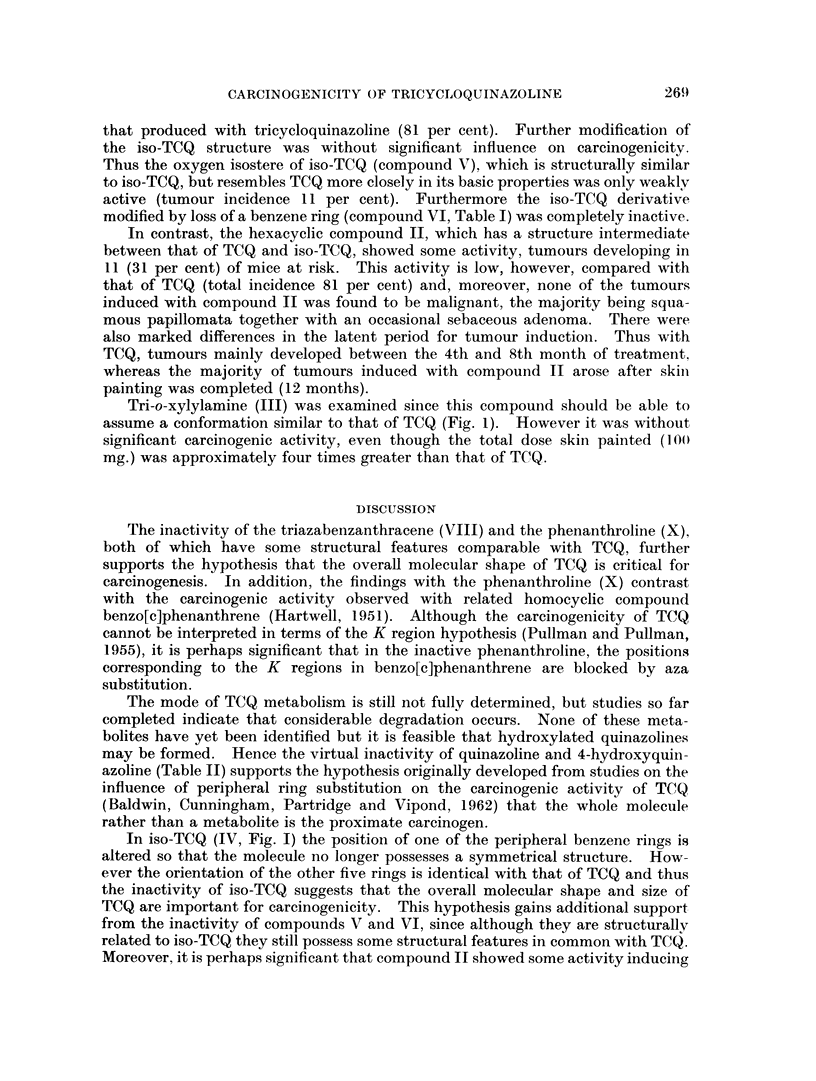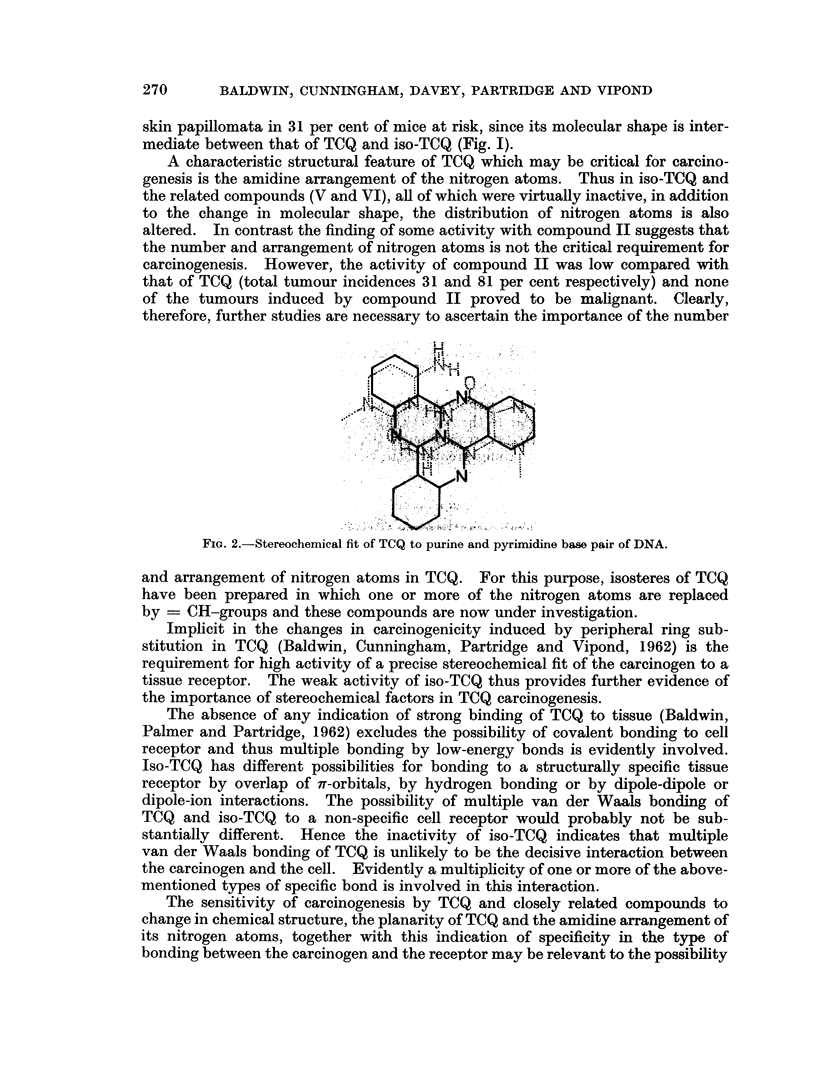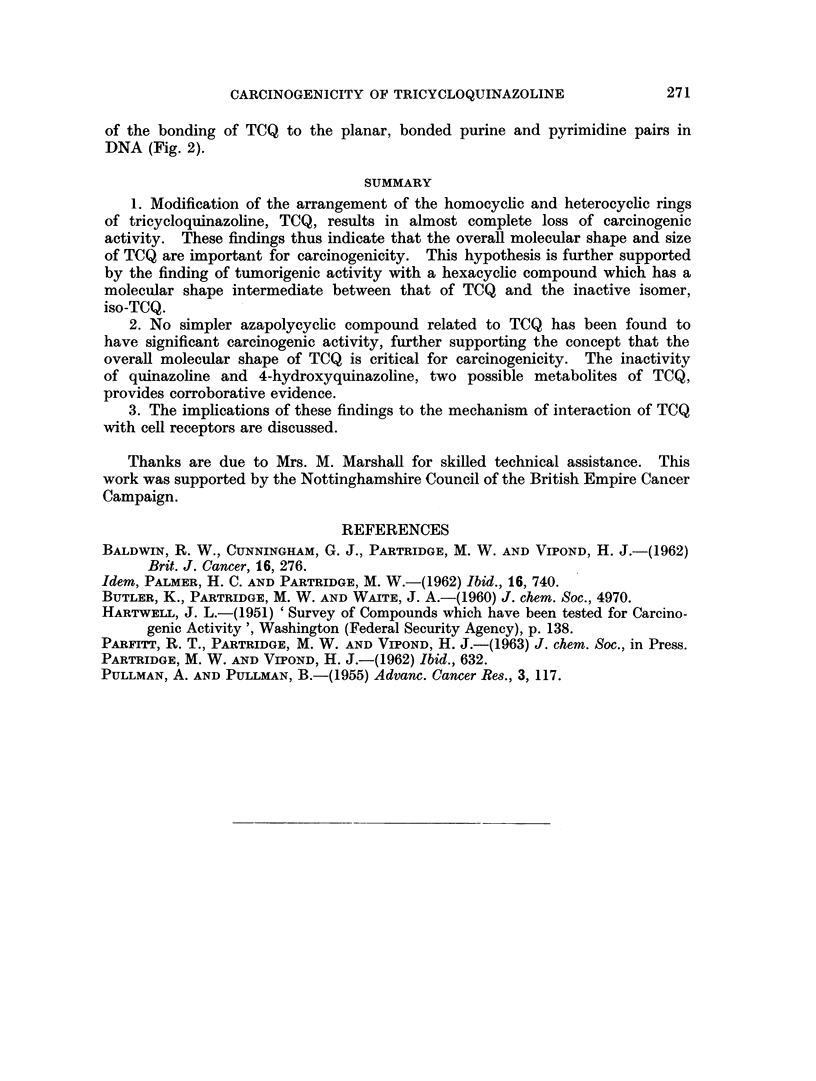# Studies on the Carcinogenicity of Tricycloquinazoline: The Effect of Structural Modification

**DOI:** 10.1038/bjc.1963.38

**Published:** 1963-06

**Authors:** R. W. Baldwin, G. J. Cunningham, A. T. Davey, M. W. Partridge, H. J. Vipond


### STUDIES ON THE CARCINOGENICITY OF TRICYCLOQUINAZOLINE : THE EFFECT OF STRUCTURAL MODIFICATION

R. W. BALDWIN, G. J. CUNNINGHAM, A. T. DAVEY, M. W. PARTRIDGE AND H. J. VIPOND

*From the Cancer Research Laboratory and Department of Pharmaceutical Chemistry, University of Nottingham, and the Department of Pathology, Royal College of Surgeons, London*

Received for publication April 30, 1963

PREVIOUSLY it was shown (Baldwin, Cunningham, Partridge and Vipond, 1962) that substitution with methyl, hydroxy, or methoxy radicals in the 2-position of tricycloquinazoline (TCQ ; I, Fig. 1) causes almost complete loss of carcinogenic activity. These findings imply the intervention of the 2- or equivalent 7- or 12-positions of the TCQ molecule in carcinogenesis.

A direct three-point union of TCQ to a cellular receptor at these positions could occur however only by covalent bonding and thus the finding that TCQ is not strongly bound in skin (Baldwin, Palmer and Partridge, 1962) excludes this possibility.

Methyl substitution in the other three possible ring positions in TCQ, namely 1-, 3-, or 4-, only partially reduces carcinogenicity, and these derivatives possess significant activities. Introduction of additional methyl substituents at the positions equivalent to the 3- position, namely 8- or 13-, brings about a very marked decrease in activity ; thus both 3,8-dimethyl-TCQ and 3,8,13-trimethyl-TCQ have very low activity. These findings suggest that stereochemical factors are important and it is considered that the evidence relating structure to activity in TCQ can be most logically accommodated in terms of the precision of stereochemical fit of the planar carcinogen by multiple low-energy bonding to a planar cell receptor site. In order further to assess the importance of molecular shape for carcinogenicity, the effect of variations in the arrangement of the homocyclic and heterocyclic rings of TCQ have been examined. The analogues tested for carcinogenic activity following skin painting in mice included an isomer, iso-TCQ (IV, Fig. 1) in which the position of one benzene ring is altered so that the molecule possesses neither a symmetrical structure nor the same distribution of nitrogen atoms as in TCQ. This compound is basic and accordingly was examined as the free base and as its nitrate. The hexacyclic compound (II, Fig. 1) was also tested because of its similarity to iso-TCQ in being a base and because its molecular shape is close to that of both TCQ and iso-TCQ. Furthermore, an oxygen isostere of iso-TCQ (V, Fig. 1) was examined since this compound, although structurally related to iso-TCQ, resembles TCQ more closely in its feeble basic properties.

A number of simpler azapolycyclic compounds structurally related to TCQ were examined also to assess the importance of overall molecular size for carcinogenicity. The compounds tested include a pentacyclic compound (VI, Fig.1) which is equivalent to iso-TCQ less a benzene ring and quinazoline and 4-hydroxyquinazoline since these substances may also be related to possible metabolites of TCQ.

### MATERIALS AND METHODS

*Mice.*—Stock male albino mice (Schofield strain), 6 to 8 weeks old at the start of each experiment.

*Skin painting.*—Dorsal hair was removed by clipping at the beginning of each test and subsequently when necessary. Mice were treated twice weekly for approximately 12 months and then kept under observation until tests were terminated (18 months). Mice were examined for the presence of tumours at weekly intervals and were killed when it was considered that tumours were malignant or when they were ill. The position of all tumours was then recorded and

Fig. 1.—Tricycloquinazoline, structural analogues and related compounds.

specimens of skin, skin tumour and other organs showing gross pathological change were taken for histological examination.

### *Details of compounds*

The azapolycyclic compounds tested all bore certain structural resemblances to known carcinogens, as illustrated in Fig. 1. Thus compounds II to VI are evidently structurally similar to TCQ (I), whilst the triazabenzanthracene (VIII) resembles a 10- substituted 1,2-benzanthracene (VII). Similarly the phenanthroline (X) resembles 3,4-benzophenanthrene (IX), whereas quinazoline (XI) and 4-hydroxyquinazoline (XII) could be related to possible metabolites of TCQ.

The syntheses and properties of these derivatives have been described previously (Butler, Partridge and Waite, 1960 ; Parfitt, Partridge and Vipond, 1963 ; Partridge and Vipond, 1962). For carcinogenic assay, all but iso-TCQ nitrate were dissolved in double re-distilled, AR grade benzene. Because of its unfavourable solubility in benzene, iso-TCQ nitrate was skin painted in 1 : 1v/v aqueous acetone. Details of the total doses and concentrations at which the compounds were tested are shown in Table I.

TABLE I.—*Details of Compounds Examined*

| Compound | Concentration tested mg./ml. | Total dose (mg.) |
|---|---|---|
| Tricycloquinazoline (TCQ) | 1·0 | 24 |
| Compound II | 1·0 | 30 |
| Tri-*o*-xylylamine (III) | 5·0 | 100 |
| Iso-TCQ. Free Base (IV) | 1·0 | 30 |
| Iso-TCQ Nitrate (in aqu. acetone) | 1·0 | 31 |
| Compound V | 1·0 | 33 |
| Compound VI | 1·0 | 31 |
| Triazabenzanthracene (VIII) | 2·5 | 53 |
| Phenanthroline (X) | 1·0 | 30 |
| Quinazoline (XI) | 5·0 | 100 |
| 4-Hydroxyquinazoline (XI) | 1·0 | 30 |

RESULTS

The incidences of skin tumours following application of tricycloquinazoline, structural analogues and related compounds to mouse skin are summarized in Table II.

TABLE II.—*Skin Tumour Incidences in Mice Treated with Tricycloquinazoline, Structural Analogues and Related Compounds*

| Compound | Number of mice at risk | Duration of experiment (days) | Total tumours | | Skin carcinomas | |
|---|---|---|---|---|---|---|
| | | | Number | Percentage | Number | Percentage |
| TCQ (I) | 36 | 471 | 29 | 81 | 27 | 75 |
| Compound II | 37 | 556 | 11 | 31 | 0 | 0 |
| Tri-*o*-xylylamine (III) | 27 | 419 | 1 | 4 | 0 | 0 |
| Iso-TCQ (IV) Free Base | 36 | 558 | 2 | 6 | 0 | 0 |
| Iso-TCQ Nitrate | 44 | 595 | 6 | 14 | 2 | 5 |
| Compound (V) | 38 | 534 | 4 | 11 | 1 | 3 |
| Compound (VI) | 37 | 488 | 0 | 0 | 0 | 0 |
| Compound(VIII) | 37 | 609 | 1 | 3 | 1 | 3 |
| Phenanthroline (X) | 37 | 556 | 2 | 5 | 0 | 0 |
| Quinazoline (XI) | 35 | 488 | 3 | 9 | 0 | 0 |
| 4-Hydroxyquinazoline (XII) | 38 | 486 | 2 | 5 | 0 | 0 |
| Benzene (Control) | 58 | 518 | 4 | 7 | 4 | 7 |

These results clearly indicate that modification of the symmetrical structure of TCQ greatly reduces carcinogenic activity. Thus iso-TCQ base proved to be virtually inactive, tumours developing in only 2 mice (6 per cent). The response elicited by iso-TCQ nitrate in aqueous acetone was slightly greater, tumours developing in 6 mice (14 per cent), but this tumour incidence is low compared to that produced with tricycloquinazoline (81 per cent). Further modification of the iso-TCQ structure was without significant influence on carcinogenicity. Thus the oxygen isostere of iso-TCQ (compound V), which is structurally similar to iso-TCQ, but resembles TCQ more closely in its basic properties was only weakly active (tumour incidence 11 per cent). Furthermore the iso-TCQ derivative modified by loss of a benzene ring (compound VI, Table I) was completely inactive.

In contrast, the hexacyclic compound II, which has a structure intermediate between that of TCQ and iso-TCQ, showed some activity, tumours developing in 11 (31 per cent) of mice at risk. This activity is low, however, compared with that of TCQ (total incidence 81 per cent) and, moreover, none of the tumours induced with compound II was found to be malignant, the majority being squamous papillomata together with an occasional sebaceous adenoma. There were also marked differences in the latent period for tumour induction. Thus with TCQ, tumours mainly developed between the 4th and 8th month of treatment, whereas the majority of tumours induced with compound II arose after skin painting was completed (12 months).

Tri-*o*-xylylamine (III) was examined since this compound should be able to assume a conformation similar to that of TCQ (Fig. 1). However it was without significant carcinogenic activity, even though the total dose skin painted (100 mg.) was approximately four times greater than that of TCQ.

### DISCUSSION

The inactivity of the triazabenzanthracene (VIII) and the phenanthroline (X), both of which have some structural features comparable with TCQ, further supports the hypothesis that the overall molecular shape of TCQ is critical for carcinogenesis. In addition, the findings with the phenanthroline (X) contrast with the carcinogenic activity observed with related homocyclic compound benzo[c]phenanthrene (Hartwell, 1951). Although the carcinogenicity of TCQ cannot be interpreted in terms of the *K* region hypothesis (Pullman and Pullman, 1955), it is perhaps significant that in the inactive phenanthroline, the positions corresponding to the *K* regions in benzo[c]phenanthrene are blocked by aza substitution.

The mode of TCQ metabolism is still not fully determined, but studies so far completed indicate that considerable degradation occurs. None of these metabolites have yet been identified but it is feasible that hydroxylated quinazolines may be formed. Hence the virtual inactivity of quinazoline and 4-hydroxyquinazoline (Table II) supports the hypothesis originally developed from studies on the influence of peripheral ring substitution on the carcinogenic activity of TCQ (Baldwin, Cunningham, Partridge and Vipond, 1962) that the whole molecule rather than a metabolite is the proximate carcinogen.

In iso-TCQ (IV, Fig. I) the position of one of the peripheral benzene rings is altered so that the molecule no longer possesses a symmetrical structure. However the orientation of the other five rings is identical with that of TCQ and thus the inactivity of iso-TCQ suggests that the overall molecular shape and size of TCQ are important for carcinogenicity. This hypothesis gains additional support from the inactivity of compounds V and VI, since although they are structurally related to iso-TCQ they still possess some structural features in common with TCQ. Moreover, it is perhaps significant that compound II showed some activity inducing skin papillomata in 31 per cent of mice at risk, since its molecular shape is intermediate between that of TCQ and iso-TCQ (Fig. I).

A characteristic structural feature of TCQ which may be critical for carcinogenesis is the amidine arrangement of the nitrogen atoms. Thus in iso-TCQ and the related compounds (V and VI), all of which were virtually inactive, in addition to the change in molecular shape, the distribution of nitrogen atoms is also altered. In contrast the finding of some activity with compound II suggests that the number and arrangement of nitrogen atoms is not the critical requirement for carcinogenesis. However, the activity of compound II was low compared with that of TCQ (total tumour incidences 31 and 81 per cent respectively) and none of the tumours induced by compound II proved to be malignant. Clearly, therefore, further studies are necessary to ascertain the importance of the number

FIG. 2.—Stereochemical fit of TCQ to purine and pyrimidine base pair of DNA.

and arrangement of nitrogen atoms in TCQ. For this purpose, isosteres of TCQ have been prepared in which one or more of the nitrogen atoms are replaced by = CH–groups and these compounds are now under investigation.

Implicit in the changes in carcinogenicity induced by peripheral ring substitution in TCQ (Baldwin, Cunningham, Partridge and Vipond, 1962) is the requirement for high activity of a precise stereochemical fit of the carcinogen to a tissue receptor. The weak activity of iso-TCQ thus provides further evidence of the importance of stereochemical factors in TCQ carcinogenesis.

The absence of any indication of strong binding of TCQ to tissue (Baldwin, Palmer and Partridge, 1962) excludes the possibility of covalent bonding to cell receptor and thus multiple bonding by low-energy bonds is evidently involved. Iso-TCQ has different possibilities for bonding to a structurally specific tissue receptor by overlap of $\pi$-orbitals, by hydrogen bonding or by dipole-dipole or dipole-ion interactions. The possibility of multiple van der Waals bonding of TCQ and iso-TCQ to a non-specific cell receptor would probably not be substantially different. Hence the inactivity of iso-TCQ indicates that multiple van der Waals bonding of TCQ is unlikely to be the decisive interaction between the carcinogen and the cell. Evidently a multiplicity of one or more of the above-mentioned types of specific bond is involved in this interaction.

The sensitivity of carcinogenesis by TCQ and closely related compounds to change in chemical structure, the planarity of TCQ and the amidine arrangement of its nitrogen atoms, together with this indication of specificity in the type of bonding between the carcinogen and the receptor may be relevant to the possibility of the bonding of TCQ to the planar, bonded purine and pyrimidine pairs in DNA (Fig. 2).

SUMMARY

1. Modification of the arrangement of the homocyclic and heterocyclic rings of tricycloquinazoline, TCQ, results in almost complete loss of carcinogenic activity. These findings thus indicate that the overall molecular shape and size of TCQ are important for carcinogenicity. This hypothesis is further supported by the finding of tumorigenic activity with a hexacyclic compound which has a molecular shape intermediate between that of TCQ and the inactive isomer, iso-TCQ.

2. No simpler azapolycyclic compound related to TCQ has been found to have significant carcinogenic activity, further supporting the concept that the overall molecular shape of TCQ is critical for carcinogenicity. The inactivity of quinazoline and 4-hydroxyquinazoline, two possible metabolites of TCQ, provides corroborative evidence.

3. The implications of these findings to the mechanism of interaction of TCQ with cell receptors are discussed.

Thanks are due to Mrs. M. Marshall for skilled technical assistance. This work was supported by the Nottinghamshire Council of the British Empire Cancer Campaign.

### REFERENCES

BALDWIN, R. W., CUNNINGHAM, G. J., PARTRIDGE, M. W. AND VIPOND, H. J.—(1962) *Brit. J. Cancer*, **16**, 276.

*Idem*, PALMER, H. C. AND PARTRIDGE, M. W.—(1962) *Ibid.*, **16**, 740.

BUTLER, K., PARTRIDGE, M. W. AND WAITE, J. A.—(1960) *J. chem. Soc.*, 4970.

HARTWELL, J. L.—(1951) ' Survey of Compounds which have been tested for Carcinogenic Activity ', Washington (Federal Security Agency), p. 138.

PARFITT, R. T., PARTRIDGE, M. W. AND VIPOND, H. J.—(1963) *J. chem. Soc.*, in Press.

PARTRIDGE, M. W. AND VIPOND, H. J.—(1962) *Ibid.*, 632.

PULLMAN, A. AND PULLMAN, B.—(1955) *Advanc. Cancer Res.*, **3**, 117.